# Stedman’s Dictionary

**Published:** 1914-12-15

**Authors:** 


STEDMAN'S DICTIONARY. The third edition of this deservedly popular Medical Dictionary is just from the press of Wm. Wood & Company. Like its predecessors it is a well made volume and a credit to the publishers. The present edition has been thoroughly revised and some thirty pages of additional matter is incorporated. This has been made necessary because of the constantly accumulating medical nomenclature. It is curious how a language grows; in two year's time sufficient additions to our medical language has accumulated to necessitate the thorough revision of a dictionary. This is one of the best medical dictionaries we have and we are glad that it is being kept up to date. Its editor, Dr. Thomas L. Stedman, has been the editor of the *Medical Record* for many years and out of his every day needs he has made a working dictionary that is all that the most exacting writers and scientific workers can hope for. This dictionary, while keeping in sight always the true etymological form of its vocabulary also defines all traditional terms in the popular form. It is a most complete and satisfactory work, and we are under obligations both to the editor and publishers for such a useful and creditable dictionary. The book may be had through the book stores, dental supply houses, or of the publishers, 51 5th Ave. N. Y., at $5.00 net.

